# 3D Evaluation of Palatal Rugae in Identical Twins

**DOI:** 10.1155/2017/2648312

**Published:** 2017-05-22

**Authors:** Emiliya Taneva, Carla Evans, Grace Viana

**Affiliations:** Department of Orthodontics, College of Dentistry, University of Illinois at Chicago, Chicago, IL, USA

## Abstract

The study of identical twins can point out potential limitations in biometrics and forensic odontology. This case report presents three-dimensional (3D) palatal rugae analysis in monozygotic twins utilizing digital models obtained directly by scanning the maxillary dental arch with the iTero® intraoral digital scanner. The results show that the rugae patterns contain related but not identical features between the pair of identical twins. Dental study models taken on a regular basis for diagnosis and treatment planning in dentistry include the palatal rugae, which could be valuable to forensics in identical twin identification cases.

## 1. Introduction

Palatal rugae, also known as plicae palatinae transversae and rugae palatine, are situated in the anterior third of the hard mucosal palate on the roof of the mouth. They appear towards the third month of intrauterine life from connective tissue covering the palatine processes of the maxillary bones. It has been shown in the literature that the palatal rugae are unique and permanent for each person and could be used for human identification [1–11]. Scanning three-dimensional (3D) technology facilitates the computerized matching of palatal rugae patterns in a manner comparable to the current gold standard for assessing fingerprints [12–14]. 3D digital models have been proven as an effective tool in evaluating palatal rugae patterns for human verification and identification [12, 15–18].

There are two basic types of twins: dizygotic (DZ), commonly referred to as fraternal twins, and monozygotic (MZ), referred to as identical twins. A higher concordance rate in MZ twins than in DZ twins has been observed.

Identical twins develop from a single zygote that splits into two individual cells and develops into two individuals. The frequency of monozygotic twins is about 0.4% across different populations [19]. A dramatic increase in the overall twinning rate has been seen, from 1 in 60 births in 1980 to about 1 in 30 births in 2013. In 2014, there were 135,336 twin births in the United States [20]. Elevated occurrence of the monozygotic twinning rate and increase of the identical twin population have been associated with medically assisted reproduction (MAR) over the past decades [21]. Biometric technologies based on different characteristics such as fingerprints, retina, face, iris, and palm prints have been developed and implemented. Identical twins share the same genetic expression, but not all biometric authentication systems provide successful verification information [22, 23]. Studying additional biometric traits such as the palatal rugae pattern to differentiate between identical twins is an important focus in biometrics and forensic odontology [14].

This case report aims to evaluate the palatal rugae pattern in a pair of identical twins, to assess the related rugae features, and to 3D-compare rugae target points with previously published values using stereolithography technology. Several studies have shown the clinical significance of the individual palatal outlines; however, 3D analysis and matching procedure in identical twins have not been previously investigated.

## 2. Materials and Methods

### 2.1. Study Design

**Figure 1:**
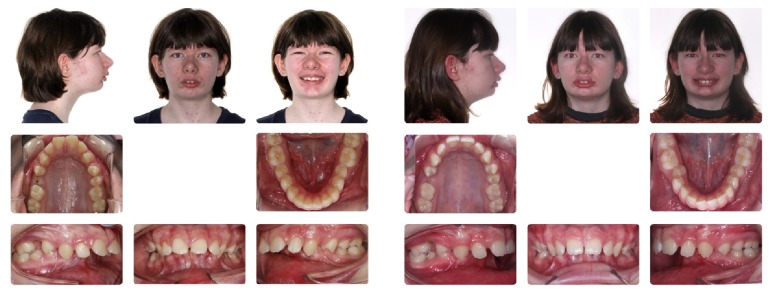
Facial and intraoral photographs of the identical twins.

**Figure 2:**
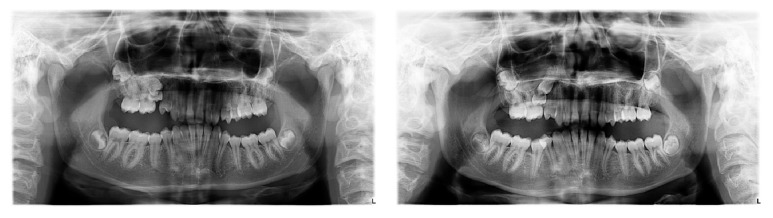
Panoramic radiographs of the identical twins.

**Figure 3:**
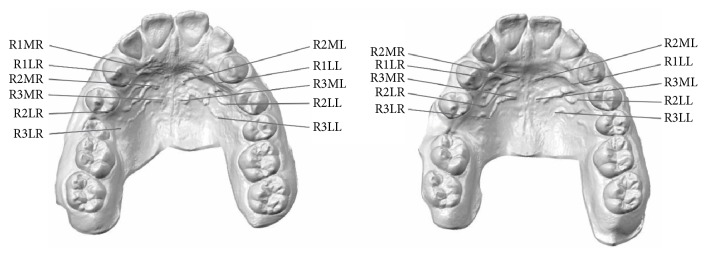
Maxillary occlusal digital models of the identical twins with the selected medial and lateral points of the palatal rugae.

A 14-year-and-10-month-old female pair of identical twins presented for an initial orthodontic visit. Facial and intraoral photographs, panoramic and lateral cephalometric radiographs, and virtual dental impressions were taken prior to orthodontic treatment (Figures 1 and 2). 3D digital models of the upper and lower jaws were obtained with the iTero HD 2.9 intraoral digital scanner (Align Technology, Inc., San Jose, CA) (Figure 3) [17, 18].

### 2.2. Palatal Rugae Assessment

Stereolithography is a typical modality of rapid prototyping used for producing physical models, patterns, and production parts in a layer-by-layer manner from computer-aided design software (CAD) via 3D printing. The iTero intraoral digital scanner employs the stereolithography apparatus technology to produce digital models derived from their data [12, 16–18]. In this case report, the identical twin patients were intraorally scanned with the iTero and digital models were exported into a stereolithography binary format (^*∗*^.stl) through the MyAlignTech website..stl is an open, industry-standard file format widely used for additive manufacturing and across different 3D modeling interfaces. The assessment, selection, and extraction of the palatal area as well as the 3D superimposition and matching process of the rugae were conducted using the.stl files imported into the professional widely used engineering processing software Geomagic® Control 14, Geomagic (Research Triangle Park, NC, USA).

The palatal rugae were documented based on their length and shape according to the Lysell, Thomas and Kotze, and Trobo classifications [8, 24, 25]. They were measured in a straight line between the origin and termination and divided into primary (with lengths of 5 mm or more), secondary (with lengths from 3 to 5 mm), and fragmentary (with lengths from 2 to 3 mm) rugae. The rugae were also categorized based on their shape as straight, wavy or sinuous, curved, and circular.

### 2.3. Palatal Superimposition

**Figure 4:**
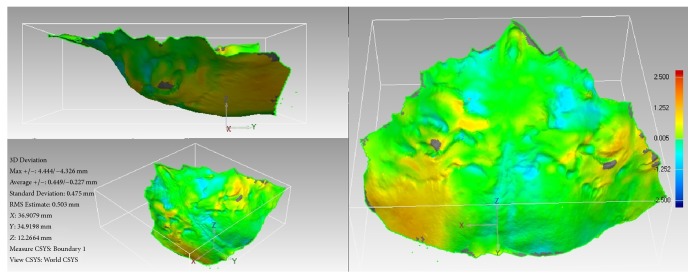
Color-coded map generated following 3D Compare analysis.

Processing and analysis of the 3D dental models were done in a set of two. Each digital impression was aligned at the same position and orientation according to the 3D coordinates (e.g., *X*, *Y*, and *Z* coordinates). The palatal rugae area of each model was selected and a separate object was extracted consisting only of that area. Manual alignment and global registration functions superimposed and fine-tuned the position of the two scans. 3D Compare analysis was performed which generated a 3D, color-coded map of the differences between the two palates (Figure 4).

**Figure 5:**
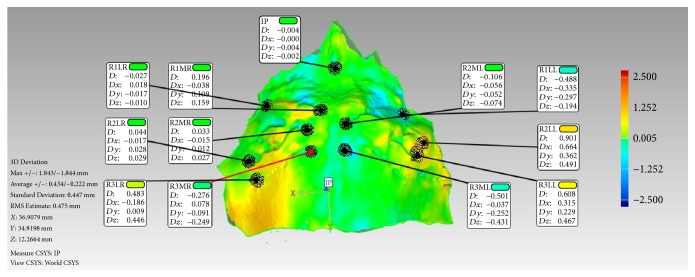
Annotation view with selected medial and lateral points of the palatal rugae.

3D surface features were identified using eleven target points: the most medial and lateral end points of the palatal rugae (R1MR, R1LR, R1LL, R2MR, R2ML, R2LR, R2LL, R3MR, R3ML, R3LR, and R3LL). Only 2 medial end points were observed for the palatal rugae on the left side. The deviations for each of the three* XYZ* coordinate axes* Dx*,* Dy*, and* Dz* and the overall deviation magnitude values for the eleven variables were automatically calculated and recorded in the Geomagic software and then exported into Microsoft® Excel (Figure 5).

The overall deviation magnitude values of the palatal rugae landmarks were compared with values previously published in the literature utilizing the same methodology. The values for the pair of identical twins were compared with the values for the same individuals over time and following orthodontic treatment and with the values for different individuals [10, 11].

### 2.4. Statistical Analysis

A data set with the eleven variables was created for this study and compared with previously published values. Descriptive and comparative statistics were performed using SPSS 22.0 (Chicago, IL). One-sample *t*-tests were used to evaluate mean discrepancies for the eleven variables in both groups. A *p* value of less than 0.05 was used as a criterion for statistical significance.

## 3. Results

### 3.1. Association of Different Rugae Lengths and Shapes between the Identical Twins

The palatal rugae length and shape were documented for both twins. Both twins were missing the first palatal rugae on the left side; one of the twins was missing the first palatal rugae on the right side as well.  Table 1 summarizes the descriptive results.

### 3.2. Comparison between Identical Twins and Same Individual Values

A one-sample *t*-test was performed to compare the mean magnitude of deviation for each of the eleven variables between the pair of identical twins iTero scans and the previously published mean magnitude of deviation for the 24 same individual's scans taken at two time periods, 20 to 24 months apart [10, 11].  Table 2 summarizes the descriptive statistics and the test results.

The results indicated that the following variables showed statistically significant mean differences: R1MR, R3MR, R2ML, R3ML, R3LR, R1LL, R2LL, and R3LL, with the *p* values ranging from 0.029 to <0.001.

The Kolmogorov-Smirnov and Shapiro-Wilk tests showed that all variables have normal distribution for the different data sets.

### 3.3. Comparison between Identical Twins and Different Individual Values

A one-sample *t*-test was performed to compare the mean magnitude of deviation for each of the eleven variables between the pair of identical twins iTero scans and the previously published mean magnitude of deviation for the 28 different individual's scans [10, 11].  Table 3 summarizes the descriptive statistics and the test results.

The results indicated that the following variables showed statistically significant mean differences: R1LL, R2LL, and R3LL, with the *p* values ranging from 0.036 to <0.001.

## 4. Discussion

Palatal rugae are considered a focus of interest and reference landmarks in dentistry, orthodontics, and forensics due to their uniqueness, stability over time, and postmortem preservation [10, 11, 25, 26]. The rugae patterns have been studied between different ethnicities, different individuals, and edentulous cases and following orthodontic treatment with expansion or extractions utilizing intraoral inspection, impressions, plaster casts, digital models, digital photography, and stereophotogrammetry [4, 5, 9]. It has also been documented that 93% of burn victims and 77% of human cadavers had no surface changes when remains were kept for a minimum period of 7 days [26]. The present case report aimed to evaluate the rugae pattern in identical twins, to determine the prevalence of similar features, and to compare the matching process with values previously published in the literature utilizing digital dental models obtained directly with the iTero intraoral scanner.

When comparing the identical twin values with the previously published data for other individual's longitudinal values, statistically significant differences were seen for eight out of the eleven variables: R1MR, R3MR, R2ML, R3ML, R3LR, R1LL, R2LL, and R3LL. When comparing the identical twin values with the previously published data for different individual values, statistically significant differences were seen for only three out of the eleven variables: R1LL, R2LL, and R3LL. The same three variables showed significant differences in both test groups. These results indicate that the monozygotic twin rugae patterns are not identical with each other and their differences are greater than individual changes seen in the reference group over time [10, 11].

A considerable correlation has been shown in fingerprint minutiae features, ridge count, ridge depth, and ridge separation in identical twins. Fingerprints of identical twins have significant generic similarity with some variations based on the micro details which are used for identification purposes [21]. Furthermore, tooth size has been suggested to have a strong hereditary component, with a trend for greater concordance in dental dimensions between monozygotic twins in comparison to dizygotic twins [27]. Experimental results have also indicated that although there is extra similarity and correlation between genetically identical vein patterns, they are distinguishable [28]. Palm prints have also demonstrated genetically related principal lines as well as some portion of weak lines for classifying identical twins [22]. Those findings are comparable with the results in this study. Correlations between the rugae lengths and shapes were observed between the identical twin pair. Both twins demonstrated the same shapes for all rugae except for the second rugae on the left side. Two of the rugae in both twins exhibited a definite continuous ring on the same location. Rugae lengths showed near-identical measurements for both twins.

This case report has assessed the palatal rugae among a pair of identical twins and has established baseline data for a larger-scale study that could be used for future comparative purposes in identical and/or fraternal twins and siblings. A longitudinal data analysis of rugae changes through time in a larger sample of multiple subjects of identical and/or fraternal twins could provide interesting results and improve the statistical power. An automated process and specialized computerized algorithm could also standardize the matching process, decrease human interaction in measurements, and increase the speed and accuracy of the quantitative analysis in large samples.

## 5. Conclusion

Digital models are taken on a daily basis in dentistry and orthodontics for records, restorative treatment, clear aligner treatment, retainer fabrication, and indirect bonding. Dental models are integrated in the personal electronic health record and can be requested by forensic institutes and law enforcement. This case report has shown that palatal rugae pattern has related but not identical features in a pair of monozygotic twins and a rugae evaluation could be a further reliable guide to forensic identification in identical twin cases.

## Figures and Tables

**Table 1:** Rugae size and shape of the identical twins pair.

Palatal landmarks	Twin 1		Twin 2	
	Size	Shape	Size	Shape
R1R	10.9 mm	Straight	Missing	Missing
R2R	12.6 mm	Circle	13.2 mm	Circle
R3R	13.1 mm	Sinuous	11.0 mm	Sinuous
R1L	Missing	Missing	Missing	Missing
R2L	10.6 mm	Sinuous	12.2 mm	Straight
R3L	13.5 mm	Circle	13.2 mm	Circle

**Table 2:** One-sample *t*-test results from the deviation magnitude comparison of identical twins and same individual values.

3D measurements	*N*	Mean (dev.)	(±) SD	*t*	df	Sig. (2-tailed)^*∗*^	95% confidence interval of the difference	
							Lower	Upper
R1MR	24	.035	.338	−2.324	23	.029	−.303	−.018
R2MR	24	−.059	.240	−1.876	23	.073	−.193	.009
R3MR	24	−.067	.249	4.113	23	.000	.104	.314
R2ML	24	.050	.265	2.891	23	.008	.045	.268
R3ML	24	−.009	.375	6.429	23	.000	.334	.650
R1LR	24	−.058	.419	−.363	23	.720	−.208	.146
R2LR	24	−.097	.484	−1.427	23	.167	−.345	.063
R3LR	24	.005	.358	−6.535	23	.000	−.628	−.326
R1LL	24	−.023	.343	6.649	23	.000	.321	.610
R2LL	24	.032	.437	−9.740	23	.000	−1.054	−.684
R3LL	24	−.028	.458	−6.798	23	.000	−.830	−.442

^*∗*^Statistically significant differences at *p* ≤ 0.05.

**Table 3:** One-sample *t*-test results from the deviation magnitude comparison of identical twins and different individual values.

3D measurements	*N*	Mean (dev.)	(±) SD	*t*	df	Sig. (2-tailed)^*∗*^	95% confidence interval of the difference	
							Lower	Upper
R1MR	28	.151	.566	−.417	27	.680	−.264	.175
R2MR	28	−.095	.762	−.888	27	.382	−.424	.168
R3MR	28	−.248	.918	.162	27	.872	−.328	.384
R2ML	28	−.108	1.037	−.012	27	.991	−.404	.400
R3ML	28	−.211	1.172	1.308	27	.202	−.165	.744
R1LR	28	−.047	1.057	−.102	27	.920	−.430	.390
R2LR	28	.140	1.318	.385	27	.703	−.415	.607
R3LR	28	.163	1.540	−1.099	27	.282	−.917	.277
R1LL	28	−.069	1.006	2.202	27	.036	.028	.809
R2LL	28	−.015	1.325	−3.657	27	.001	−1.430	−.402
R3LL	28	−.195	1.534	−2.772	27	.010	−1.398	−.209

^*∗*^Statistically significant differences at *p* ≤ 0.05.
